# Elucidation of the molecular responses during the primary infection of wild blueberry phenotypes with *Monilinia vaccinii-corymbosi* under field conditions

**DOI:** 10.1186/s12870-021-03281-2

**Published:** 2021-10-27

**Authors:** Sherin Jose, Joel Abbey, Laura Jaakola, David Percival

**Affiliations:** 1 Wild Blueberry Research Program, Faculty of Agriculture, Dalhousie University, Truro, NS B2N 5E3 Canada; 2 Climate laboratory Holt, Department of Arctic and Marine Biology, The Arctic University of Norway, NO-9037 Tromsø, Norway; 3 NIBIO, Norwegian Institute of Bioeconomy Research, P.O. Box 115, NO-1431 Ås, Norway

**Keywords:** Monilina blight, Gene expression, Pathogenesis-related protein, Wild blueberry, Flavonoid pathway genes

## Abstract

**Background:**

Monilinia blight caused by *Monilinia vaccinii-corymbosi* (Reade) Honey (*M.vc*) is a major disease of wild blueberry that can result in severe crop losses in the absence of an integrated disease management programme. The fungus causes blight in the emerging floral and vegetative buds, but the degree of susceptibility varies among the different wild blueberry phenotypes, ranging from the highly susceptible *V. a.* f. *nigrum* to the moderately susceptible *V. angustifolium* and the least susceptible *V. myrtilloides*.

**Results:**

The present study evaluated the defense responses of these major phenotypes during their primary infection (floral buds) with *M.vc*. The temporal expression profiles of PR genes (*PR3* and *PR4*) and the flavonoid pathway structural genes (*CHS*, *ANS*, *ANR*, *DFR* and *FLS*) were analysed. The *PR3* and *PR4* gene expression profiles revealed that *V. myrtilloides* responded to *M.vc* infection by activating the expression of both PR genes. *V. a.* f. *nigrum,* on the other hand, failed to activate these genes, while *V. angustifolium*, exhibited an intermediate response. Our study with the flavonoid pathway genes indicated variability in activation of the genes during post-infection time points with *ANS* and *ANR* in *V. myrtilloides*, *FLS* in *V. angustifolium* and no response observed in *V. a.* f. *nigrum*.

**Conclusions:**

Altogether, this study highlights that the degree of phenotype susceptibility is associated with the timely activation of host defense responsive genes. Data obtained in this study provided a starting point for a better understanding of the wild blueberry- *M. vaccinii-corymbosi* pathosystem.

**Supplementary Information:**

The online version contains supplementary material available at 10.1186/s12870-021-03281-2.

## Background

Wild blueberry, also known as the lowbush blueberry, is a woody perennial of the family *Ericaceae* [1] native to the Atlantic Provinces of Canada and Maine, US. Wild blueberries are unique and differ from highbush blueberries in terms of their origin, climate and species involved. Most commercial wild blueberry fields are developed by removing overstory vegetation from forested areas and scrublands having wild blueberry rhizomes [2]. Given the native nature of the plants, commercial fields are typically made up of clonal patches of the wild blueberry phenotypes. Among the phenotypes on commercial fields, *Vaccinium angustifolium* (tetraploid) its subspecies (*V. a*. f. *nigrum*) form 70–80% on a surface area basis whereas *V. myrtilloides* (diploid) form ~ 10–20% [3, 4].

Due to the increasing interest in food aspects related to human health benefits, the interest, production and consumption of blueberries are increasing because of the abundance of phenolic compounds and associated antioxidant capacity. Wild blueberries are known to be one of the richest sources of anthocyanins and other flavonoids [5]. An increasing body of evidence suggests the beneficiary roles of anthocyanins in health which includes scavenging free radicals, anti-inflammatory and antimicrobial action, improvements in memory and cognitive performance and cardiovascular health [5, 6]. Given the increasing knowledge on the dietary and nutritional composition of blueberries, there has been a growing demand for their consumption in the last decades [7–9].

Despite the commodity’s importance and rising demand, its production is faced with many challenges including fungal diseases. Monilinia blight is a commercially damaging disease on wild blueberry fields and is caused by *Monilinia vaccinii-corymbosi* (Reade) Honey (*M.vc*), which also attacks almost all *Vaccinium* spp. [10, 11]. The infection cycle starts early spring with the release of ascospores from mummified berries, which infects budding floral and vegetative buds, culminating in blight (primary infection) [12]. Infected leaves appear water-soaked, and turn dark brown, beginning at the base and progressing along the midrib and veins of leaves, which quickly wilt [10, 12]. Individual blossoms and clusters brown and wither, but remain attached to the plant. Although difficult to see, the fungus appears on the infected leaf midrib and at the base of blossoms as a white-greyish mass of spores. Mummy berries are formed when conidia grown on these blighted tissues infect the flowers’ ovaries (secondary infection) [13]. Infected fruit shrivels, hardens, and turns salmon in colour several weeks before harvest [12]. The disease can be destructive under favourable weather conditions such as prolonged wetness [14, 15], resulting in significant losses in berry yield and post-harvest quality [2, 16]. As documented by Hildebrand and Braun [17], Monilinia blight of emerging leaf and floral buds (primary infection) causes large yield losses in lowbush blueberry, whereas, yield losses are more affected by mummy berries in highbush blueberry [18]. Based on field observations, Monilinia blight management is quite challenging, as fungicides have become the sole economically viable option [10, 19, 20]. However, with the progressive restriction in the use of conventional fungicides, studying the plants’ natural resistance could be an effective disease management strategy.

Generally, plants in the field are continually subjected to a multitude of stresses and in the case of wild blueberries; they are in constant exposure to disease pressures due to their native and unique growing conditions and maritime climate. Furthermore, most efforts to genetically elucidate the Monilinia-blueberry pathosystem have focused solely on highbush blueberry cultivars or other *Vaccinium* spp. [21], with no attempt to comprehend the molecular responses of wild blueberry phenotypes to Monilinia blight (primary infection) to yet. A stepping stone for improving our understanding of the responses of wild blueberry- Monilinia pathosystem would be to analyze the expression of pathogenesis-related genes (*PR3* and *PR4*) and flavonoid pathway genes (*CHS*, *ANS*, *ANR*, *DFR* and *FLS*). The PR proteins can respond to both biotic and abiotic stresses and belongs to different classes as described by van Loon et al. [22]. Numerous studies have described the selective expression of PR-protein encoding genes following infection with a wide range of pathogens, whether it is necrotrophic or biotrophic [23–26]. According to Piasecka et al. [27] certain defensive secondary metabolites are strongly induced after pathogen infection. Among these, flavonoids are the most important in wild blueberries and several studies have reported that the flavonoid components accumulate to act as chemical messengers, physiological regulators and inhibitors against phytopathogenic organisms [28–30]. Therefore, they may have the potential to protect plants from phytopathogens.

In the present study, we compared the molecular responses of the three major wild blueberry phenotypes after challenging them with *Monilinia vaccinii corymbosi* under field conditions. Their levels of defense response were analyzed in a time course pattern and compared to an uninfected control. Yield parameters and harvestable berry yield of each phenotype were also analyzed after harvest.

## Results

### Monilinia blight infection in wild blueberry phenotypes

**Fig. 1:**
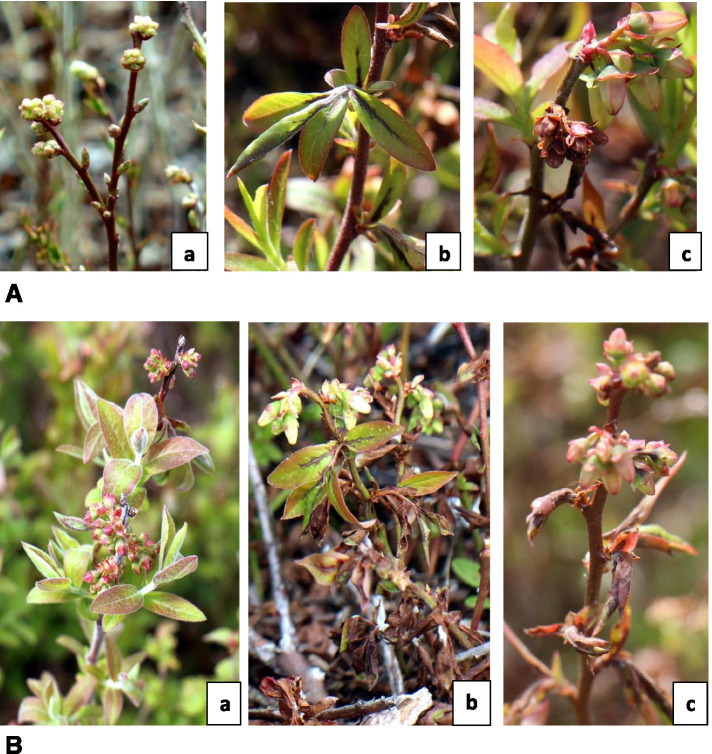
**A**. Floral bud stage and Monilinia blight symptoms. **a** Floral bud at F3 stage (Monilinia susceptible stage, sepal covered individual flowers are visible (Annis, 2009)) (**b**) Infected leaf turn dark brown starting at the base along the midrib and veins and (**c**) Infected blossoms turn brown and wither but remain attached to the plant. **B.** Phenotypic variations in wild blueberry phenotypes in response to *M.vc* infection at 6 dpi (days post infection). **a** *V. myrtilloides*; **b** *V. angustifolium* and **c** *V. a*. f. *nigrum*

Wild blueberry phenotypes were monitored for Monilinia blight symptoms after artificial *M.vc* inoculation at the F3 stage (Fig. 1A) of floral bud growth under field conditions. Because of the phenotypes’ variability in floral and vegetative bud emergence, symptoms first appeared on *V. a*. f. *nigrum* (Supplementary file Fig. 1). Infected leaves turned to dark brown starting from the base along to the midrib and veins, while infected blossoms turned dark purple-brown (Fig. 1A- b & c) but remain attached to the plant. Both *V. angustifolium* and *V. a*. f*. nigrum* exhibited noticeable blossom and leaf blight by 6 dpi (days post inoculation), where the floral buds at the F5/6 stage (Fig. 1B). By direct observation, the disease severity on *V. a*. f*. nigrum* was higher than *V. angustifolium*. However, the symptoms were not apparent in *V. myrtilloides* at both 6 and 10 dpi with only a weak infection of leaf tissues and no infections of floral clusters.

### Pathogenesis-related gene responses in wild blueberry phenotypes during *M.vc* infection

**Fig. 2:**
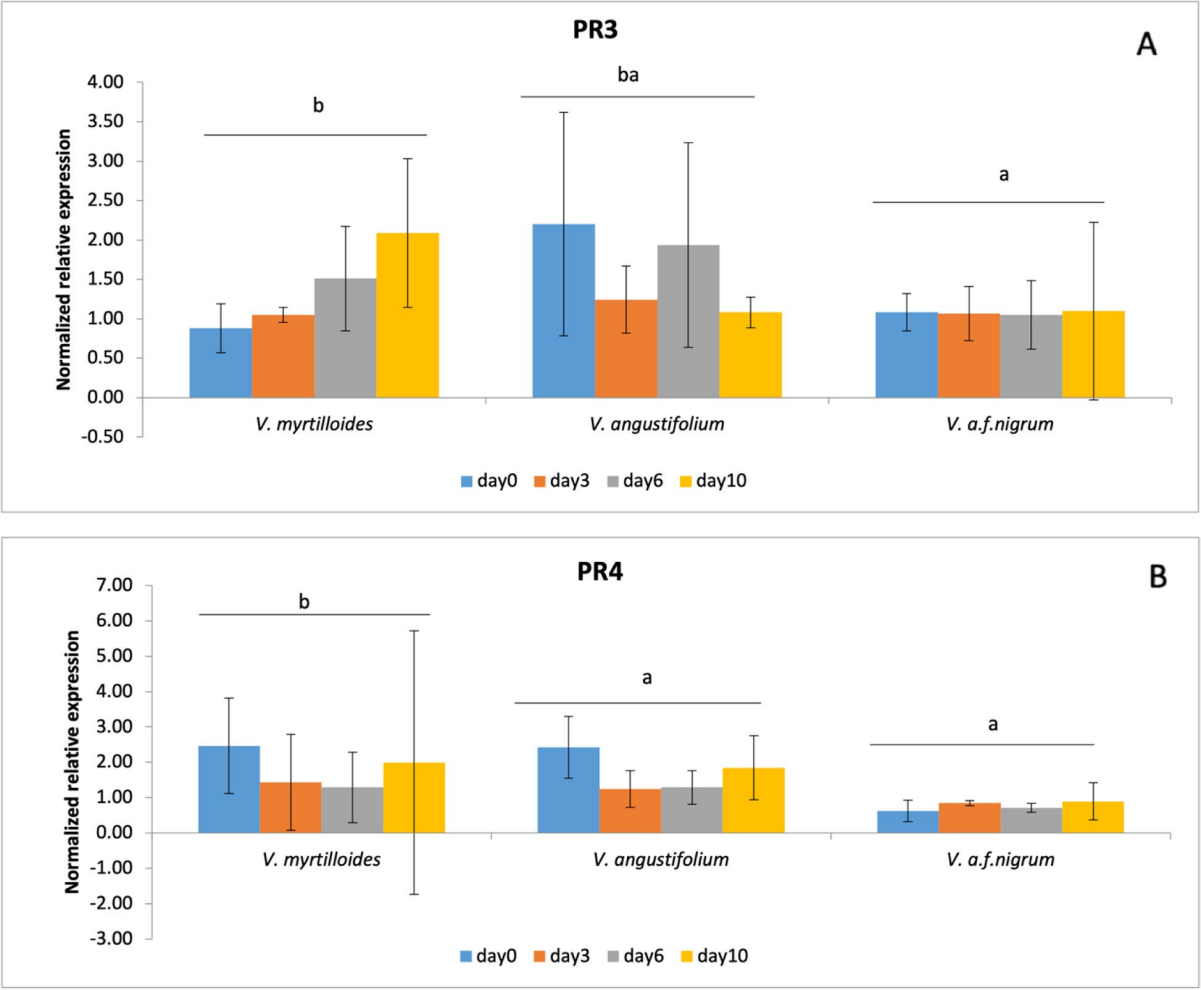
Relative expression profiles of PR3 and PR4 in wild blueberry phenotypes- *V. myrtilloides*, *V. angustifolium*, and *V. a*. f. *nigrum* in response to *Monilinia vaccinii-corymbosi* infecton. **A** *PR3* (pathogenesis-related gene 3) and **B** *PR4* (pathogenesis-related gene 4)**.** Expression of each gene is shown as–fold change relative to the untreated control from the same time point. Error bars represent the mean ± SD of *n* = 3 biological replicates, 15 stems per replicate. Phenotypes with same letters are not significantly different from each other at α = 0.05 using the PROC GLIMMIX procedure of SAS

The temporal expression pattern of pathogenesis-related genes *PR3* and *PR4* were analyzed in wild blueberry phenotypes under field conditions. These genes were evaluated for basal expression (day 0) and 3, 6 and 10 dpi (days post-infection) by using quantitative PCR. The basal transcript levels of both PR genes exhibited differential induction between the phenotypes (Fig. 2). *V. angustifolium* exhibited the highest level of basal expression for both PR genes, whereas no response was observed for *V. a.* f. *nigrum*. The time-course expression study revealed that *PR3* (2.20 fold at 10 dpi) and *PR4* (2.08 fold at 10 dpi) were significantly up-regulated in *V. myrtilloides* after *M.vc* infection. A gradual increase through the time points was observed and reached higher expression at 10 dpi. However, for *V. angustifolium* the highest induction was detected in *PR3* (1.70 fold) at 6 dpi and followed by a steady decrease, whereas for *PR4* the maximum peak (1.84 fold) occurred at 10 dpi. Strikingly, the expression of *PR3* was not induced in *V. a.* f. *nigrum* compared to the control condition whereas down-regulation was detected for *PR4*. *V. myrtilloides*, the most tolerant phenotype responded to *M.vc* infection by inducing both PR genes. On the contrary, *V. a.* f. *nigrum* the highly susceptible phenotype was unable to activate such responses.

### Expression profiles of flavonoid biosynthesis pathway genes

**Fig. 3:**
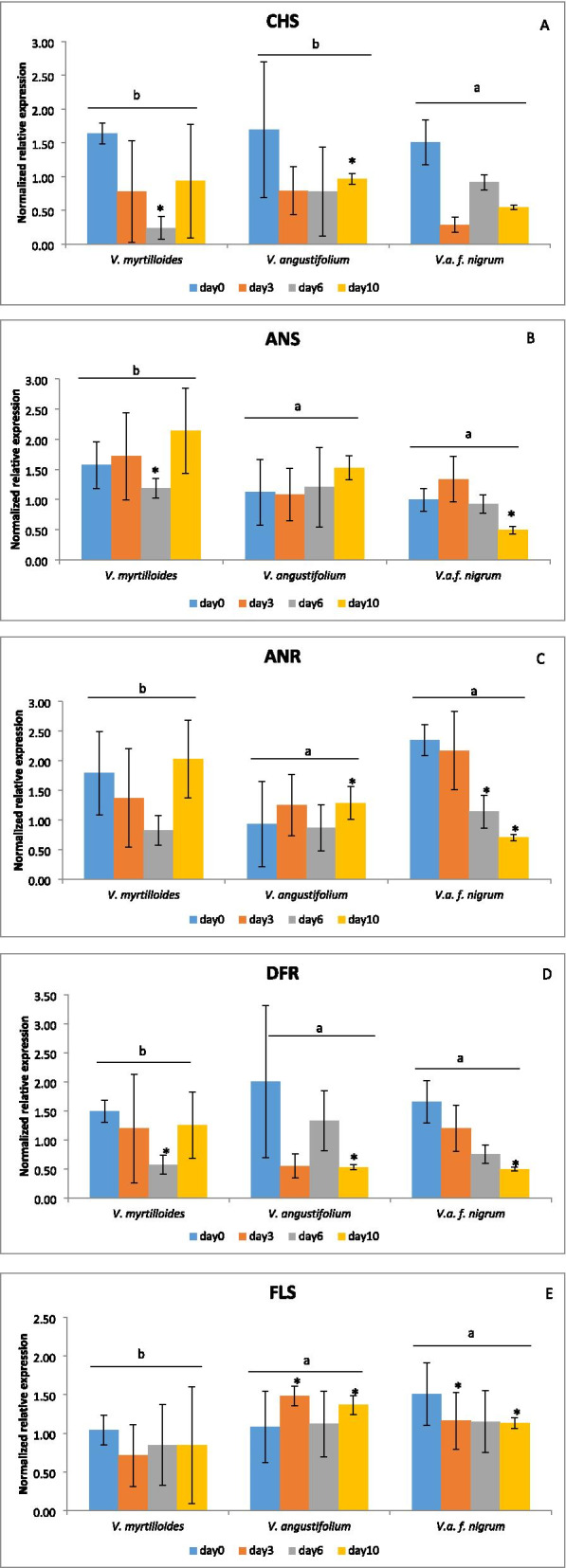
Relative expression profiles of Flavonoid biosynthesis pathway genes in *V. myrtilloides, V. angustifolium*, and *V. a*. f. *nigrum* in response to *Monilinia vaccinii-corymbosi* infecton. **A** Chalcone synthase (*CHS*)**; B** Anthocyanin synthase (*ANS*)*;* **C** Anthocyanin reductase (*ANR*); **D** Dihydroflavonol-4-reductase (*DFR*); and **E** Flavonol synthase **(***FLS)***.** Expression of each gene is shown as–fold change relative to their respective untreated control from the same time point. Error bars represent the mean ± SD of *n* = 3 biological replicates, 15 stems per replicate. When comparing fold changes among the three phenotypes, those with same letters are not significantly different from each other at α = 0.05. The asterisks indicate significant difference compared with the different days of inoculation using the PROC GLIMMIX procedure of SAS

The expression of key structural genes related to the flavonoid biosynthesis pathway was analyzed in wild blueberry phenotypes in response to *M.vc* infection (Fig. 3). *CHS*, *DFR*, *ANS*, *ANR* and *FLS* were the genes studied (Fig. 3). The *CHS* gene expression pattern was found to be relatively consistent across the phenotypes. All the three phenotypes showed a basal induction (1.64, 1.50 and 1.69-fold respectively), followed by down-regulation at the respective post-infection time-points (Fig. 3a). In the case of *ANS*, higher expression was observed for *V. myrtilloides* at 10 dpi (2.14-fold) and *V. angustifolium* also expressed a slight increase for the *ANS* gene at 10 dpi (1.53-fold). However, in *V. a*. f. *nigrum* the highly susceptible phenotype, *ANS* gene showed lower expression for the studied time-points (Fig. 3b) compared to other species. *ANR* showed higher expression in *V. a*. f. *nigrum* at day 0 (2.35-fold) and 3 dpi (2.17-fold) but it was later downregulated at 6 dpi and 10 dpi. Interestingly, *V. myrtilloides* showed higher expression at 10 dpi (2.03-fold) and *V. angustifolium* showed a discrepancy in expression for all time points (Fig. 3c). *V. angustifolium* expressed the highest peak for *DFR* gene at the basal level when compared with the other two phenotypes. However, all the three phenotypes showed a discrepancy in expression for the post-infection time-points (Fig. 3d). *FLS* expression was found to be a bit higher for *V. angustifolium* at post-infection time-points and for *V. a.* f. *nigrum* the highest peak was observed at basal level only (day 0, 1.51-fold). No remarkable change in expression for *FLS* gene was observed in *V. myrtilloides* (Fig. 3e). When comparing the phenotypes and the different days post infection, no statistically significant interaction was observed for any of these genes, but significance was observed for some time-points within the phenotypes. However, it should be noted that significance was observed between the most tolerant and the susceptible phenotypes with most of the analyzed genes.

### Wild blueberry phenotypes yield parameters

Wild blueberry phenotypes were analysed for different yield components (set fruit and pinhead) and harvestable berry yield (Table 1). A significant treatment effect was observed with all the yield parameters. *V. myrtilloides* exhibited the highest pinhead when compared to other phenotypes and treatments. Although the fruit set was significant, most of the treatments did not vary significantly from each other except un-inoculated *V. angustifolium* had the least fruit set. There was also a significant yield difference among the treatments. The un-inoculated *V. angustifolium* and its subspecies f. *nigrum* had the highest yield compared to their *M.vc* treated ones. Interestingly, the *M.vc* treated *V. myrtilloides* also had a significantly higher yield.

**Table 1:** Assessment of the yield parameters (set fruit, pinhead) and harvestable berry yield among wild blueberry phenotypes

Phenotype	Treatment	No. of set fruit	No. of pinhead	Berry yield (g.m-2)
*V. myrtilloides*	Control	4.93 ± 0.69a	7.00 ± 0.68a	364.78b
	M.vc treated	3.88 ± 0.75ba	3.35 ± 0.76b	563ba
*V. angustifolium*	Control	1.90 ± 0.94b	3.02 ± 0.81b	525.89ba
	M.vc treated	4.86 ± 1.39a	3.94 ± 1.19b	279.67b
V.a. f. nigrum	Control	6.31 ± 0.98a	4.07 ± 0.87b	757.44a
	M.vc treated	5.38 ± 0.77a	2.93 ± 0.65b	250b
ANOVA results ^a^		*P* = 0.0465	*P* = 0.0298	*P* = 0.0341

^a^Analysis of variance (ANOVA) results refer to treatment effects that were either not significant (NS) or significant at *p* < 0.05. Mean separation was completed using LSD test procedure (ά = 0.05). Means in a column with the same letters are not significantly different from each other

## Discussion

Wild blueberry fields are extremely heterogeneous and structured as mosaic patches of phenotypically diverse clones such as *V. angustifolium* Aiton, *V. angustifolium* f. *nigrum* Wood and *V. myrtilloides* Michx [31, 32]. The phenotypes can be distinguished from each other by differences in flower, stem and leaf colour and shape, plant height, developmental phenology and berry colour [10, 33]. The variability exhibited by the wild blueberry phenotypes can also be correlated to its varied defense response machinery. The present study was undertaken to analyze the molecular responses of the wild blueberry phenotypes to *Monilinia vaccinii-corymbosi* infection under field conditions. Research on the variations in phenotypic responses to *M.vc* should aid in the effective management of Monilinia blight in the field. This study represents the first investigation of gene expression analysis in wild blueberry- *M.vc* primary infection and provides additional evidence for the varied resistance/susceptibility response between the phenotypes.

The variability in disease incidence and severity observed among phenotypes after *M.vc* inoculation agrees with the severity of Monilinia blight described by Lockhart et al. [16]. The infections were more severe on *V. a.* f. *nigrum* than *V. angustifolium* and with less or no infection on *V. myrtilloides*. Previous field studies reported a positive correlation between the bud development stages during ascospore release [14, 17]. We observed an earlier vegetative and reproductive bud break in *V. a.* f. *nigrum* than the other studied phenotypes (Supplementary Fig. 1). Ehlenfeldt & Stretch [34] compared the highbush and rabbiteye blueberry cultivars resistance to Monilinia leaf blight and found that cultivars with earlier shoot growth had a considerably higher percentage of blighted shoots than other cultivars. The studies on the variations in the severity of mummy berry disease in high bush blueberry cultivars [35] and low bush blueberry clones [2] indicate that plants can avoid infection by having little or no susceptible tissue during the ascospore release. Although avoidance due to delayed floral/vegetative bud development is likely an important Monilinia blight resistance mechanism, however, the variations in host response might be investigated in the absence of this mechanism. As a result, in the present study, we inoculated the floral buds for all the phenotypes at the same developmental stage (F3 stage) and analysed the defense response of individual genes over time.

The present study demonstrated contrasting expression levels of PR genes between the tolerant *V. myrtilloides* and the highly susceptible *V. a*. f. *nigrum*, with *PR3* and *PR4* gene activation observed in the tolerant phenotype. *PR3* and *PR4* are chitinases, which inhibit fungal growth by degrading chitin present in their cell walls [22, 26]. Several studies have reported increased expression of multiple PR genes during biotic stress [36, 37]. Susceptibility, according to van Loon [22], corresponds not only to a lack of the required defense machinery but also to the delayed activation of the pathogen-fighting genes. In *V. myrtilloides*, *PR3* exhibited a gradual upregulation with time-points after infection, however, *V. a.* f. *nigrum* had no response suggesting that a lack of response could be the explanation of its high susceptibility (Fig. 2A). Conversely, *V. angustifolium* exhibited a discrepancy in expression with high up-regulation of *PR3* before infection and reduced expression at 3 dpi. This lack of early response (3 dpi) could be a partial reason why it is not resistant to *M.vc*. Research has shown that the *PR2*, *PR3* and *PR10* genes are repressed in susceptible highbush blueberry cultivar after infection with *Colletotrichum acutatum* [24]. In our study, the *PR4* gene expression also resulted in an induced expression in *V. myrtilloides* followed by *V. angustifolium* but repression in *V. a*. f. *nigrum* (Fig. 2B). Several studies [22, 23, 38] reported PR gene repression including *PR3* and *PR4* genes as an indication of a reduction in the plant’s self-defense mechanism, thereby facilitating the progression of the infection process within the plant. We observed repression of both the *PR3* and *PR4* genes in the highly susceptible phenotype, *V. a*. f. *nigrum* and a discrepancy in expression in *V. angustifolium* suggesting its moderate degree of susceptibility.

Being a managed crop in its natural habitat, wild blueberry plants cannot circumvent environmental stressors. Many biochemical pathways are adaptable to meet plants’ environmental responsiveness [39]. Several studies suggest that flavonoid biosynthesis play an important role in plant defense machinery against biotic stress by the accumulation of flavonoid components [36, 40, 41]. In the present study, we evaluated the expression of flavonoid biosynthesis pathway structural genes such as *CHS*, *ANR*, *ANS*, *DFR* and *FLS* in response to *M.vc* inoculation. The *CHS* gene, which initiates the flavonoid biosynthesis pathway, is induced in plants under a variety of biotic and abiotic stress conditions [42, 43]. In contrast, *CHS* showed a high basal expression in all the wild blueberry phenotypes followed by repression post-infection (Fig. 3a). Interestingly, the expression of downstream flavonoid structural genes differed in expression between the phenotypes. Based on their level of defense, the phenotypes may have differentially manipulated the transcription mechanism responsive to *M.vc* infection. According to our findings, *V. myrtilloides*, the most tolerant phenotype responded to *M.vc* infection by activating *ANS* and *ANR* at 10 dpi only (Fig. 3b &c). This can be correlated to the phenotype’s disease resistance capacity, as observed in the field study (Fig. 1B-a). In contrast, no notable expression of the ANS gene was observed in *V. a*. f. *nigrum*, while *ANR* showed induction at the basal level (day 0) and during the early infection phase (3 dpi). In *V. a*. f. *nigrum*, the expression of most of the flavonoid structural genes was highest at the basal stage only (day 0), pointing towards the lack of gene activation during post-infection in this phenotype. Based on flavonoid accumulation, Lu et al. [28] reported distinct resistance responses of two apple cultivars to rust infection. Metabolic analyses focusing on this group of metabolites might be needed to confirm the induction of this pathway in each phenotype - *M.vc* interaction.

Overall, the present study found that in response to Monilinia blight, there are differential expressions of defense-related genes between the wild blueberry phenotypes with clear induction of several genes only in *V. myrtilloides*, the tolerant phenotype. Therefore, it may be hypothesized that the differences in response observed between the three phenotypes could be explained, at least partly, by the differential expression of antifungal defense genes and the activation of the flavonoid biosynthesis pathway genes. The study is a first step towards the understanding of defense activation in wild blueberry phenotypes.

### Experimental procedures

#### Plant material and experimental design

Clonal patches of the wild blueberry phenotypes- *V. myrtilloides, V. angustifolium* f. *nigrum* and *V. angustifolium* were selected from a commercial wild blueberry field, NS, Canada. Wild blueberry fields are part of native vegetation and are commercially managed crops in their natural habitat. The plant materials were collected in compliance with institutional and national guidelines [44]. The study was supported by Bragg Lumbar Company and the Wild Blueberry Producers Association of Nova Scotia and permission is not required for sample collection.

*V. myrtilloides* (diploid) is tolerant to Monilinia blight whereas *V. angustifolium* and *V. angustifolium* f. *nigrum* (tetraploid) are susceptible and highly susceptible phenotypes respectively [16]. Three biological replicates were selected for each phenotype and each replicate was separated into two, 0.5 × 1 m sample areas. The experiment began when 80% of the floral buds per phenotype reached the F3 stage (floral bud scale separation and appearance of new growth) [19]. For *V. a.* f. *nigrum* and *V.angustifolium*, inoculation performed on May 30, 2019 and for *V. myrtilloides*, it was on June 11, 2019. One day before inoculation, one sample area within each replicate was sprayed with the fungicide Proline® (a.i. prothioconazole) at a rate of 315 ml product·ha^− 1^ using a CO_2_ powered, Bell spray Inc. hand-held research sprayer with 2 m boom with 4 Tee Jet Visiflow 8002VS nozzles at a pressure of 220 kpa to serve as treated/control plots. In addition, a Watchdog (Spectrum Technologies) weather station was placed in the field equipped with temperature, relative humidity and leaf wetness sensors that recorded environmental data at 15 min intervals throughout the season.

### Fungal culture and plant inoculation

*Monilinia vaccinii-corymbosi* cultures were isolated from mummy berries and Monilinia blighted shoots collected from commercial wild blueberry fields in Nova Scotia during 2018. Tiny blocks of white medulla cut from the center of the surface-sterilized mummy berries and blighted leaf tissues were placed on potato dextrose agar (PDA) (Difco) plates amended with a mixture of 0.5 mg·mL^− 1^ streptomycin sulfate and 0.5 mg·mL^− 1^ penicillin to prevent bacterial contamination [10]. All plates were placed in an incubator at 22 ± 2 °C in the dark [10] until *M.vc* colonies were observed on the medium. Sporulation was performed as per the procedure described by Guo (2016). Conidia were isolated by filtration and adjusted to a concentration of 2 × 10^5^ conidiophores·mL^− 1^ by using a hemocytometer. Each phenotype treatment group was sprayed with *M.vc* inoculum at all angles until runoff. The control group was mock-inoculated with sterile water. The sample area was immediately covered with 2 mm plastic film and row cover to provide incubating conditions (100% RH), required for Monilinia infection [45]. After 72 h, the plastic film and row cover were removed and floral bud tissue from 15 random stems in each plot (inoculated and mock-inoculated) was harvested for RNA extraction and immediately flash frozen in liquid nitrogen and stored at − 80 °C. Floral tissues were collected as day 0 (before inoculation), 3, 6 and 10 days after inoculation.

### Yield component and berry yield assessment

Ten blueberry stems were collected diagonally along a line transect in each clonal patch per phenotype to examine yield potential after the fruit set had occurred. This allowed the evaluation of set fruits and pinheads (small unmarketable berries). In addition, harvestable berry yield was determined by harvesting blueberries (late August) using a forty-tine hand rake from two randomly selected 30 × 30 cm quadrats from each control/treated patch.

### RNA extraction and cDNA synthesis

Total RNA was isolated from frozen floral buds of three biological replicates per phenotype (control and *M.vc* inoculated) using RNeasy plant mini kit (Qiagen, US). Residual genomic DNA was digested by RNase-free DNase (Qiagen, US) according to the manufacturer’s instructions. The concentration and purity of RNA samples were assessed using Nanodrop ND 1000 spectrophotometer. RNA samples with an OD260/280 value between 1.8 and 2.2 were considered as high-quality RNA. The integrity of RNA was assessed using 1.2% (w/v) agarose gel electrophoresis. Single-stranded cDNA was synthesized from 1 μg of total RNA using High Capacity cDNA Reverse Transcription Kit (Applied Biosystems) using random primers according to manufacturer’s instructions and stored at − 20 °C until use.

### Primer design

Gene-specific sequences were retrieved from *V. corymbosum* database (www.vaccinium.org). Specific primers were designed and amplified on *V. myrtilloides, V. angustifolium* f. *nigrum* and *V. angustifolium*. Amplified products were isolated and sequenced. Wild blueberry specific primers were designed and verified using different bioinformatics tools (BioEdit/ Clustal w/BLAST/ Primer Premier 5.0). Primer Premier 5.0 (Premier Biosoft International, Palo Alto, California, USA) was used to design primers suitable for qPCR analysis (Supplementary Table 1). The following parameters were chosen: primer length of 18–24 base pairs (bp), primer melting temperature (Tm) between 58 °C and 64 °C, and guanine-cytosine (GC) content of 40–60%. The amplification efficiency of each primer was calculated using a ten-fold cDNA dilution series with three replicates per concentration to generate a five-point standard curve for estimation of amplification efficiency (E = (10^[− 1/slope]^ − 1) × 100%) and correlation coefficient (R^2^).

### Quantitative real-time PCR (qRT-PCR) analysis

The qRT-PCR assay was performed using a CFX Connect Real-time PCR Detection System (Bio-Rad, CA, US). Each PCR reaction mixture (10 μl) contained 2 μl of diluted cDNA (20-fold dilution (5 ng/ μl)), 5 μl SsoAdvanced™ SYBR® Green Supermix (Bio-Rad), and 1 μl (10 nM) of each forward and reverse primer. The amplification program was as follows: an initial denaturation at 95 °C for 3 min, followed by 40 cycles at 95 °C for 10 s, 60 °C for 20 s. Each run was completed with a melting curve analysis (65–95 °C with at increments of 0.5 °C) to verify the specificity of the amplification. GAPDH was selected as the reference gene for *V. angustifolium* f. *nigrum* and *V. angustifolium* and UBC9 for *V. myrtilloides* [46]. A no-template control (NTC) was included with each run for each gene to confirm the absence of non-specific products. Three technical replicates were performed for each biological replicate in each qPCR experiment. Relative expression levels of the genes were calculated by the 2 − ^ΔΔCT^ method [47].

### Statistical analysis

The statistical analysis was carried out using the PROC GLIMMIX procedures of SAS (version 9.3, SAS Institute, Inc., Cary, NC). LSD (Least Significant Difference) was used for multiple means comparison at the level of α = 0.05.

## Supplementary Information

**Additional file 1: Table S1**. List of target genes, reference genes, specific primer sequences and supporting information used for qRT-PCR analysis to determine the expression in wild blueberry phenotypes.

**Additional file 2: Figure S1**. Variability in floral bud emergence observed among the wild blueberry phenotypes.

## Data Availability

All data that supports the findings of this study are included in the article and its supplementary information files.

## Abbreviations

ANS: Anthocyanidin synthase
ANR: Anthocyanidin reductase
CHS: Chalcone synthase
DFS: Dihydroflavonol 4-reductase
dpi: Days post infection
FLS: Flavonol synthase
GAPDH: Glyceraldehyde-3-phosphate dehydrogenase
NTC: no-template control
PR: Pathogenesis-related protein
PDA: Potato dextrose agar
qPCR: Quantitative polymerase chain reaction
UBC: Ubiquitin-conjugating enzyme S
