# Supplementary material for: Elucidation of the molecular responses during the primary infection of wild blueberry phenotypes with Monilinia vaccinii-corymbosi under field conditions
Source: BMC Plant Biol. 2021 Oct 27;21:493. doi: 10.1186/s12870-021-03281-2 (PMC8549177; doi:10.1186/s12870-021-03281-2)
Supplement: Supplementary file 1 — Additional file 1: Table S1. List of target genes, reference genes, specific primer sequences and supporting information used for qRT-PCR analysis to determine the expression in wild blueberry phenotypes. [file 12870_2021_3281_MOESM1_ESM.docx]

**Table S1.** List of target genes, reference genes, specific primer sequences and supporting information used for qRT-PCR analysis to determine the expression in wild blueberry phenotypes

| **Sl.No** | **Gene name** | **Genbank ID** | **Primer sequence (5'-3')** | **Annealing temperature** | **Amplicon size (bp)** | **Primer efficiency (%)** | **Regression coefficient (R2)** | **Reference** |
| --- | --- | --- | --- | --- | --- | --- | --- | --- |
| ***Pathogenesis-related (PR) genes*** | | | | | | | | |
| 1 | PR3 | MK292725 | F-TGTGCTCCTGGGAAGAAGTA | 55 | 112 | 100 | 0.998 | this study |
|  |  |  | R-AGTCTGGGTTGGCTAGTAGAT |  |  |  |  |  |
| 2 | PR4 | MK292723 | F-TAACTACAACCCGGAGCAGG | 56 | 164 | 102 | 0.998 | this study |
|  |  |  | R-GCAAGCACTTCCCACAAGAA |  |  |  |  |  |
| ***Flavonoid pathway structural genes*** | | | | | | | | |
| 3 | CHS | MK333526 | F-TCCCAGATCAAGAAGAGGTACA | 57 | 119 | 103 | 0.997 | this study |
|  |  |  | R-ATTTCCACAACCACCATATCCT |  |  |  |  |  |
| 4 | ANS | MK333528 | F-GAATCACCTGAGAGCCCTAAC | 58 | 75 | 102 | 0.999 | this study |
|  |  |  | R-AGCCTGTCTTCTTCCAATCC |  |  |  |  |  |
| 5 | ANR | MH321471 | F-CAAAGACCCTAGCGGAGAAAG | 56 | 98 | 97 | 0.999 | this study |
|  |  |  | R-GGAGAAACACCAGCCATAAGA |  |  |  |  |  |
| 6 | FLS | MK333530 | F-CTCCTTCTTACAGGGAAGCTAATG | 58 | 79 | 99 | 0.996 | this study |
|  |  |  | R-GACAGCCACTTGAACAACTTG |  |  |  |  |  |
| 7 | DFR | MK333524 | F-CTGCTGGAACCGTCAATGT | 58 | 139 | 101 | 0.996 | this study |
|  |  |  | R-GCTGCTTTCTCTGCTAGTGTT |  |  |  |  |  |
| ***Reference genes*** | | | | | | | | |
| 9 | GAPDH | AY123769 | F-CAAACTGTCTTGCCCCACTT | 55 | 207 | 98 | 0.998 | Koskimäki et al., 2009 |
|  |  |  | R-CAGGCAACACCTTACCAACA |  |  |  |  |  |
| 10 | UBC9 | *contig13673 | F-CACCCGAATATAAACAGCAATGG | 55 | 91 | 99 | 0.997 | this study |
|  |  |  | R-ACAGCAACACCTTGGAGATAG |  |  |  |  |  |
| *Sequence retrieved from <http://bioinformatics.towson.edu/BBGD454/> | | | | |  |  |  |  |
