# Supplementary figures and images for: Elucidation of the molecular responses during the primary infection of wild blueberry phenotypes with Monilinia vaccinii-corymbosi under field conditions
Source: BMC Plant Biol. 2021 Oct 27;21:493. doi: 10.1186/s12870-021-03281-2 (PMC8549177; doi:10.1186/s12870-021-03281-2)

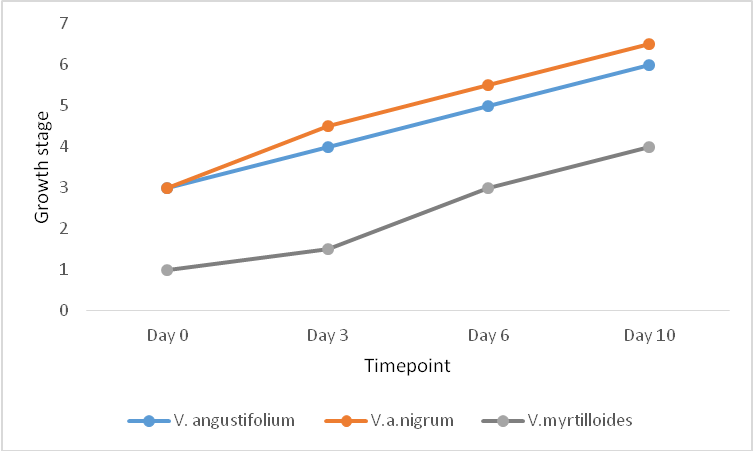

Supplement: Supplementary file 2 — Additional file 2: Figure S1. Variability in floral bud emergence observed among the wild blueberry phenotypes. [file 12870_2021_3281_MOESM2_ESM.docx]
